# Impact of the digital health application ViViRA on spinal mobility, physical function, quality of life and pain perception in spondyloarthritides patients: a randomized controlled trial

**DOI:** 10.1186/s13075-024-03443-1

**Published:** 2024-12-03

**Authors:** Paloma Palm von Alten Blaskowitz, Anna-Maria Liphardt, Claudia Bouzas, Birte Coppers, Pascal Petit, Nicolas Vuillerme, Vanessa Bundle, Sebastian Rudolf, Johannes Knitza, Maria Gabriella Raimondo, Hannah Labinsky, Lukas Hatscher, Andreas Wirsching, Daniela Bohr, Elizabeth Araujo, Andreas Ramming, Alina Ramming, Georg Schett, Harriet Morf

**Affiliations:** 1 Department of Medicine 3 - Rheumatology & Immunology, Friedrich-Alexander-Universität (FAU) Erlangen-Nürnberg and Uniklinikum Erlangen, Erlangen, Germany; 2 Deutsches Zentrum für Immuntherapie (DZI), Friedrich-Alexander-Universität Erlangen-Nürnberg and Uniklinikum Erlangen, Erlangen, Germany; 3 Univ. Grenoble Alpes, AGEIS, Grenoble, France; 4 Institut Universitaire de France, Paris, France; 5 Institute for Digital Medicine, University Hospital of Giessen and Marburg, Philipps-University Marburg, Marburg, Germany; 6 Department of Internal Medicine 2, Rheumatology/Clinical Immunology, University Hospital Würzburg, Würzburg, Germany

**Keywords:** Spondyloarthritides, Spondyloarthropathy, Psoriatic spondylitis, Digital health application, DHA, ViViRA, E-Health, Physical function, Mobility

## Abstract

**Background:**

Spondyloarthritides (SpAs) are a group of common rheumatic diseases that often cause limited mobility and lower back pain. Physiotherapy is an integral part of treatment, but access to physiotherapy limits treatment success. Digital health applications (DHAs) enable home-based physiotherapy and could significantly improve access for SpAs patients. The aim is to investigate the clinical effects of the DHA ViViRA compared with those of standard physiotherapy.

**Methods:**

SpAs patients with chronic back pain were enrolled in a randomized controlled trial. The intervention group received ViViRA DHA, whereas the control group received standard physiotherapy. Pain (verbal rating scale, PAIN-Detect), quality of life (SF-36) and mobility (BASMI) were assessed at baseline and after 12 weeks as the primary outcomes.

**Results:**

Data from 59 participants (71.2% female, mean age 45.2 years) were analyzed. The intervention group showed a significant improvement in mobility (average BASMI score: baseline: 1.1 [range 0.7–1.5]; follow-up: 1.0 [range 0.5–1.4]; *p* = 0.05), whereas the control group showed a significant decrease in mobility (baseline: 1.5 [range 1.1–1.9]; follow-up: 1.8 [range 1.4–2.2]; *p* = 0.00). The intervention group demonstrated lower pain intensity (VRS pain level at week 3.5 ± 2.8) than did the control group (VRS pain level at week 4.5 ± 2) after 12 weeks.

**Conclusion:**

Our results highlight the efficacy of DHAs such as ViViRA in the treatment of lower back pain in SpAs patients. Compared with the current gold standard, physiotherapy, DHA use results in superior outcomes. However, further larger studies are needed to confirm these promising results.

**Trial registration:**

The study is registered in the German clinical trial registry (DRKS) under the following ID: DRKS00031254.

**Supplementary Information:**

The online version contains supplementary material available at 10.1186/s13075-024-03443-1.

## Introduction

Spondyloarthritidess (SpAs) are one of the most common rheumatic diseases in Germany, with a prevalence of 0.32–0.5% [1]. It affects axial and peripheral joints and presents with sacroiliitis, enthesitis, or dactylitis [2]. Pain and joint stiffness compromise SpAs patients´ quality of life and physical function. Inflammation results in structural change in the spine and lower back pain, and alongside pharmacological treatment, physiotherapy or related nonpharmacological treatments are key for preserving physical function in patients with SpAs. Owing to the challenging and often late diagnosis, these patients do not start adequate therapy in time and physical functional limitations are often already present at a young age [3]. This makes it more important that patients with SpAs have easy access to exercise-related interventions. Considering the rapid development of digital tools that can remotely guide physical exercise, there is great potential to guide patients in disease specific home-based exercise interventions to preserve spinal mobility. Digital tools can provide better accessibility to therapy and increase physical activity through independent and flexible functional training [4].

Physical activity can reduce pain, enhance spinal mobility, and decrease functional impairment [5, 6]. A combination of cardiorespiratory fitness, muscle strength, flexibility and neuromotor performance is fundamental in the management of SpAs. Currently, physiotherapy is the gold standard of nonpharmacological treatment [7] that has been shown to reduce functional limitations in SpAs patients within 12 weeks of physiotherapy [8] and is more effective in improving spinal mobility than home-based workouts because of better adherence [8]. Previous studies have consistently demonstrated the benefits of exercise programs in patients with SpAs, including reduced pain and disease activity, increased mobility, and better physical function [9–11]. Training sessions that combined flexibility and resistance training exercises had the greatest effect on spinal mobility [12].

Digital health applications (DHAs) have the potential to overcome barriers to changing physical activity habits, as they bring exercise directly to patients’ homes. Additionally, the DHA saves time in exercising classes (64%), enables health monitoring (48.9%), and provides accurate information about diseases (40.9%) [13]. Other studies have shown promising results regarding the use of digital therapeutics for rheumatic patients’ fitness and health [14]. The vast majority of rheumatic patients (91.2%) regularly use a smartphone, and patients are eager to use digital technologies to self-manage their disease [15, 16]. Unfortunately, high-quality exercise applications tailored to SpAs patients are still lacking [17, 18]. Most of the applications aim to collect disease data: a digital therapeutic for rheumatology care is the monitoring app “Abaton”, which can record the course of therapy [19], whereas “Rheuma-Auszeit” (Rheuma-Liga, German patient organization for rheumatic diseases) provides patients with relaxation and exercise [20]. ViViRA (ViViRA Health Lab GmbH c/o Mindspace, Berlin), a German DHA, offers an innovative approach for providing personalized exercise programs to patients with nonspecific low back pain or osteochondrosis [21, 22]. The effectiveness of ViViRA in reducing pain (collected by VRS) was demonstrated in a randomized, controlled, open-label intervention study where low back pain was measured over 12 weeks while patients used ViViRA or received physiotherapy [23].

The aim of this study was to investigate the effects of 12 weeks of exercise therapy with ViViRA on spinal mobility, pain and quality of life in patients with SpAs compared with standardized physiotherapy.

## Methods

### Study design and patient recruitment

This prospective, randomized, controlled study was conducted between February 2023 and January 2024, and participants for the intervention and control group were recruited from the outpatient clinics of the Department of Rheumatology and Immunology at the Universitätsklinikum Erlangen, Germany. Male and female patients with axial spondyloarthropathy (ASAS criteria 2009) [24] or psoriatic spondylitis [25], between 18 and 70 years of age and with chronic lower back pain (numeric rating scale for pain > 4/10 for > 3 months), were included [26]. Participants had stable disease activity and had not changed medication in the last three months before inclusion in the study. The exclusion criteria were malignant bone diseases, existing limiting orthopedic diseases and pregnancy (according to the ViViRA contraindications) [27]. Compliance with the use of the application was monitored through personal conversation, and the patients received a customized feedback questionnaire after 12 weeks.

### Ethical considerations

The study protocol was approved by the medical faculty ethics committee of the Friedrich-Alexander-Universität Erlangen-Nürnberg, Erlangen, Germany (22-425-Bm) and registered in the German Clinical Trials Register (DRKS-ID DRKS00031254) [28].

Participation in the study was voluntary. All patients provided their written informed consent before study inclusion. The participants were coded with a pseudonym. The collected data were stored and analyzed in a password-protected database (REDCap), and only previously defined and authorized persons had access. Patients had the option of withdrawing their participation in the study at any time, whereby all personal data were irrevocably deleted. The study was conducted in accordance with the ethical guidelines of the Declaration of Helsinki.

### Measurements

Upon inclusion, patients were randomly assigned to one of the two groups (intervention or control group). After the patients were recruited, baseline measurements were taken, and the patients answered questionnaires. The questionnaires were collected digitally via REDCap (Research Electronic Data Capture) [29]. Sociodemographic data were collected at baseline. Clinical parameters were retrieved from the patients´ clinical records.

Spinal mobility was assessed with the Bath Ankylosing Spondylitis Metrology Index (BASMI) [0–10, where 0 is not limited and 10 highly limited] and the modified Schober´s-test (measured by tape) [30, 31].

### Patient reported outcome measures

An overview of the administered patient reported outcome measures is provided in Table 1.

**Table 1:** Questionnaires

Questionnaires	Abbreviation	Unit	Range	Measurement	References
Verbal Rating Scale	VRS	Scale	0 to 10: 0 = no pain, 10 = highest pain	Patient perceived pain in the last 7 days, generally and especially back pain	[32]
PAIN-Detect	PAIN	Score	0 to 38: 0–12 = neuropathic pain component unlikely, 13–18 = uncertain, 19–38 = neuropathic pain component probably	Detection of neuropathic pain	[33]
Short Form 36 Questionnaire	SF-36	Score	0 to 100: higher score indicates a better health status	Health-related quality of life, 36 items that assess physical and psychological health	[34]

### Digital health application

**Fig. 1:**
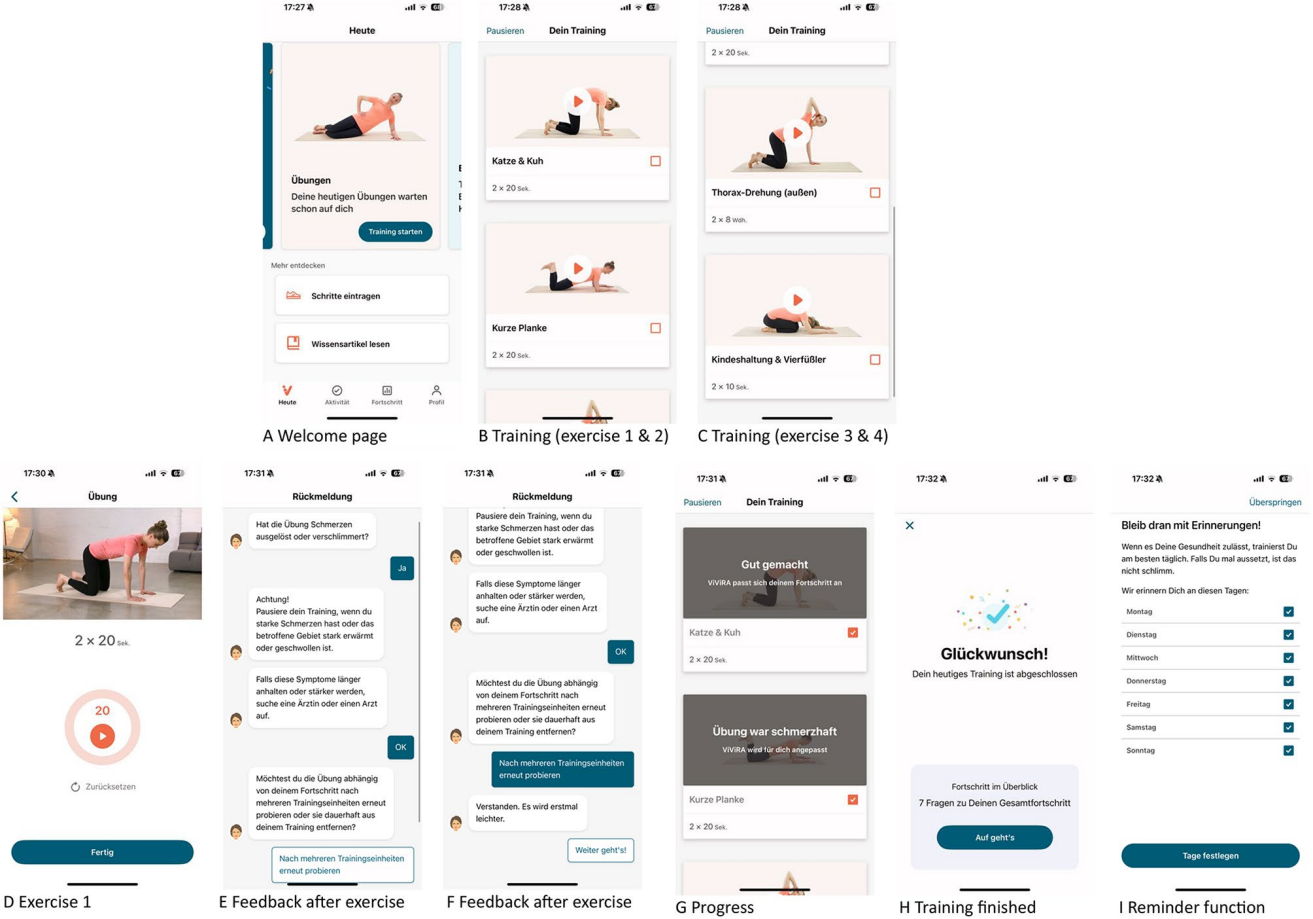
Screenshots of the DHA ViViRA

ViViRA was prescribed to the patients in the intervention group for the first time on the day of baseline data collection. After prescription, patients could download the application (app) with a code for use on a smartphone or tablet and sign an additional data protection declaration to use ViViRA [35]. The participants were instructed to exercise with ViViRA at least three times per week for 15 min and were reminded of the sessions by the ViViRA app reminder function. One session comprised 3–5 exercises (e.g., *cat and cow*, *short plank*, *thoracic rotation*, *quadruped position* and *child´s pose*, Fig. 1). After each exercise, patients were requested to provide feedback on their exercise execution, and the exercise protocol (intensity and complexity) was consequently adapted automatically by the app according to the individual fitness and pain level. Performance during the training sessions and the evolution of pain and mobility are visualized in the activity history of the app.

### Control intervention

The 29 participants in the control group were prescribed standard of care physiotherapy (once a week; approx. 30 min /session for 12 weeks). The physiotherapy sessions were not standardized, but depended on the practitioner and the patient and comprised muscle strength, flexibility, balance and neuromotor exercises. Physical activity was documented in a questionnaire.

### Statistical analysis

Statistical analyses were performed via R 4.3.1^®^ software (R Core Team, Vienna, Austria) for Windows 10©. The type I error rate was set at α = 0.05. All the results are reported as the means +/- standard deviations (SDs).

The descriptive analysis of the population was conducted for each time point (i.e., baseline and follow-up), overall, and separately for both groups (i.e., control and intervention).

For continuous variables, descriptive analysis was performed by computing the number of observations, the number of missing observations, the arithmetic means and standard deviation, the median and IQR (interquartile range), and the minimum-maximum range. For the categorical variables, the number of participants and their proportion/percentage were calculated.

Potential interaction effects between time points (i.e., baseline and follow-up) and groups (i.e., control and intervention) were examined. With respect to continuous variables, to investigate the interaction effect and examine fixed effects (between-subjects factor) and repeated measures (within-subjects factor), a two-way mixed ANOVA was used. If one of the assumptions (i.e., the assumption of normality of residuals, homogeneity of covariances, and sphericity) was violated, a robust two-way mixed ANOVA was performed. The main effects were investigated with estimated marginal means (emmeans) via pairwise comparisons. The Benjamini-Hochberg was used to account for multiple testing in pairwise comparisons. The two-way mixed ANOVA and checking of related assumption validity were carried out via the rstatix package [36], whereas robust two-way mixed ANOVA was performed via the WRS2 package [37]. Estimated marginal means (emmeans) were calculated via the emmeans package, which was also used for pairwise comparisons [38].

For nonordered categorical variables, a logistic regression analysis was performed, whereas for ordered categorical variables, an ordered logistic regression was conducted. Ordered logistic regression was performed via the MASS package [39].

## Results

### Patient characteristics

Fifty-nine patients participated in the study, and all basic characteristics are summarized in Table 2.

**Table 2:** Patient characteristics (baseline)

Patient characteristics	All patients (*n* = 59)	Intervention group (*n* = 30)	Control group (*n* = 29)	*p*-value
Age [years] *mean ± SD*	45.2 ± 11.2	43.1 ± 11.3	47.2 ± 11.1	*p* = 0.16
Sex female *number (%)*	*n* = 42 (71.2%)	*n* = 21 (70%)	*n* = 21 (72.4%)	*p* = 1.00
BMI [kg/m2] *mean ± SD*	27.2 ± 5.1	27.2 ± 5.3	27.2 ± 5.0	*p* = 0.32
BASMI *mean ± SD*	1.3 ± 1.2	1.1 ± 1.1	1.5 ± 1.3	*p* = 0.22
Modified Schober´test [cm] *mean ± SD*	12.5 ± 4.6	13.6 ± 3.9	11.4 ± 5.0	*p* = 0.07
VRS pain level of last week [0–10] VRS back pain level [0–10] *mean ± SD*	4.8 ± 2.7 4.9 ± 2.8	4.3 ± 2.9 4.7 ± 3.1	5.3 ± 2.2 5.2 ± 2.4	*p* = 0.17 *p* = 0.55

Patient characteristics of the intervention group (using ViViRA) and control group (using physiotherapy) in a randomized controlled study of 59 SpAs patients (71.2% female, 28.8% male) in Erlangen, Germany, from February 2023 to January 2024. *BMI* body mass index, *BASMI* Bath Ankylosing Spondylitis Metrology Index, *VRS* verbal rating scale

### Influence on mobility and function

**Fig. 2:**
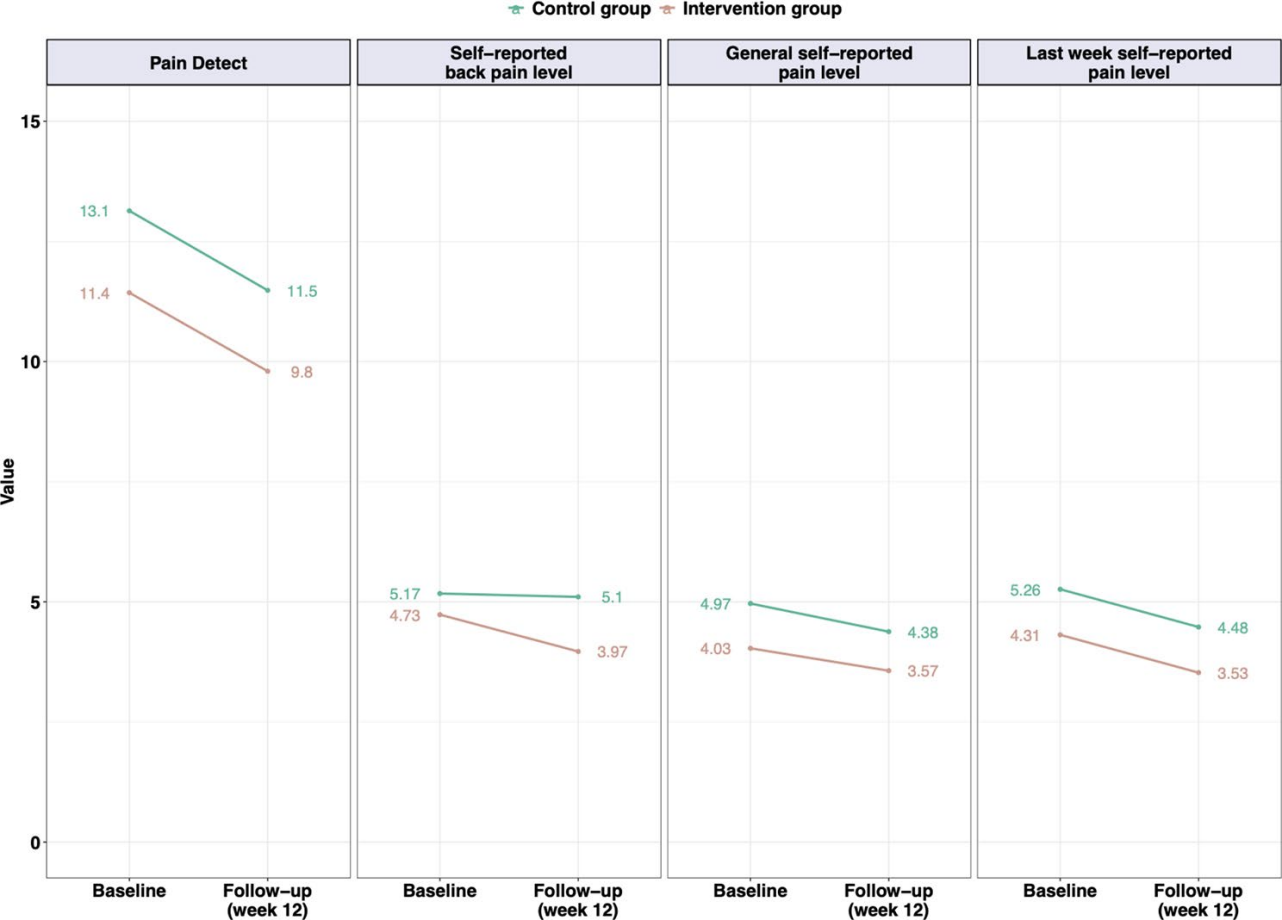
Results at baseline vs. follow-up (*mean value).* The results regarding pain perception of the intervention group (using ViViRA, *n* = 30) and control group (performing physiotherapy, *n* = 29) at baseline vs. follow-up (after 12 weeks) in a randomized controlled trial with 59 SpAs patients (71.2% female, 28.8% male)

The BASMI score was higher for the control group than for the intervention group at follow-up (Fig. 2). It was higher for females (females: 1.6 [range 1.3-2]; males: 0.7 [range 0.1–1.2]; *p* = 0.00) and older participants (< 45 years old: 0.8 [range 0.5–1.2]; ≥ 45 years old: 2 [range 1.6–2.4]; *p* = 0.00). Between baseline and follow-up, the BASMI score decreased in the intervention group (baseline: 1.1 [range 0.7–1.5]; follow-up: 1.0 [range 0.5–1.4]; *p* = 0.05), whereas it increased in the control group (baseline: 1.5 [range 1.1–1.9]; follow-up: 1.8 [range 1.4–2.2]; *p* = 0.00).

The modified Schober´s test increased in response to exercising with the ViViRA application (intervention group: baseline 14 cm [range 6–19 cm]; follow-up 16 cm [range 7–20 cm]; control group: baseline 11 cm [range 3–22 cm]; follow-up 11 cm [range 4.8–17 cm]; Fig. 2). Overall, it was greater for males (females: 12 cm [range 11–13 cm]; males: 14 [range 12–16 cm]; *p* = 0.05) and for younger participants (< 45 years old: 14 cm [range 12–15 cm]; ≥ 45 years old: 11 cm [range 9.8–13 cm]; *p* = 0.02). Regardless of the time point considered, the modified Schober´s test was greater in the intervention group than in the control group.

The quality of life according to the SF-36 physical score increased between baseline and follow-up in both groups (baseline: 38 [range 36–41]; follow-up: 41 [range 39–44]; *p* = 0.00). The quality of life was lower for females (females: 38 [range 35–40]; males: 46 [range 41–50]; *p* = 0.00) and older participants (< 45 years old: 43 [range 40–46]; ≥ 45 years old: 35 [range 32–39]; *p* = 0.05).

### Influence on pain perception

**Fig. 3:**
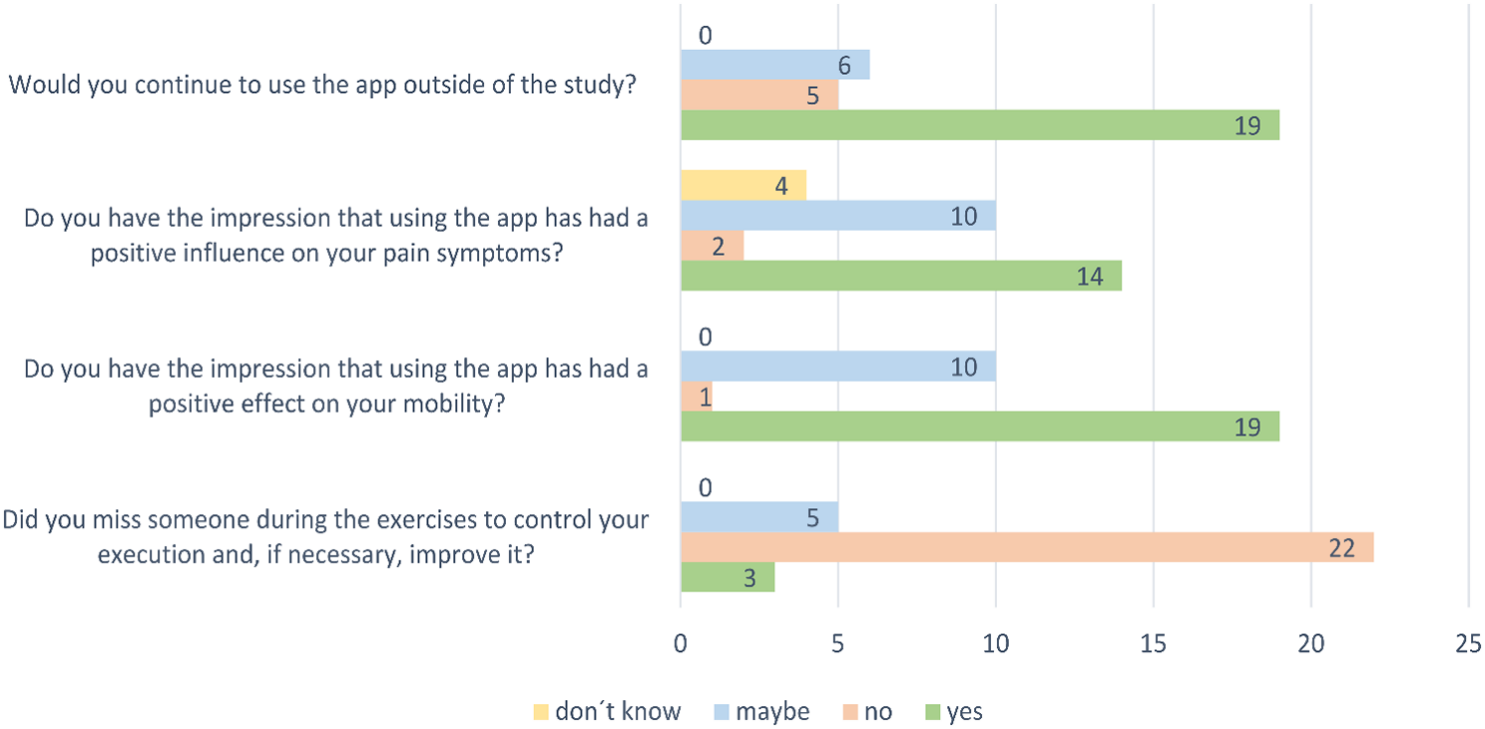
Results of the feedback questionnaire. The results of the feedback questionnaire of the intervention group (*n* = 30) after 12 weeks of exercising with ViViRA in a randomized controlled trial with 59 SpAs patients (71.2% female, 28.8% male)

**The PAIN-Detect score** was higher at baseline than follow-up in both groups (baseline: 12 [range 10–14]; follow-up: 11 [range 8.8–12]; *p* = 0.00, Fig. 3). Overall, it was especially higher for females (females: 12 [range 10–14]; males: 9.2 [range 6–12]; *p* = 0.00). Participants with fewer physical limitations (reduced HAQ score) had a reduction in the probability of neuropathic pain at follow-up (baseline: 13 [range 10–16]; follow-up: 10 [range 7.7–13]; *p* = 0.00).

There was no group effect **on self-reported general pain level during the last 7 days (by VRS)**, values decreased in both groups between baseline and follow-up (baseline: 4.8 [range 4.1–5.4]; follow-up: 4.0 [range 3.3–4.7]; *p* = 0.00; Fig. 2).

Overall, the **back pain level** was greater at baseline than at follow-up for overweight participants (BMI < 25 kg/m2: 3.7 [range 2.4-5]; BMI ≥ 25 kg/m2: 5.5 [range 4.6–6.3]; *p* = 0.03). In addition, in the overweight patients, the level of back pain was lower at follow-up than at baseline. (BMI ≥ 25 kg/m2 at baseline: 5.5 [range 4.6–6.3]; follow-up 4.5 [range 3.7–5.4]; *p* = 0.01; BMI < 25 kg/m2 at baseline: 3.7 [range 2.4-5]; follow-up 4.5 [range 3.2–5.8]; *p* = 0.14).

### Feedback questionnaire ViViRA

The self-reported training adherence of the participants in the intervention group (*n* = 30) is summarized in Table 3. All the results of the feedback questionnaire after the training with ViViRA are shown in Supplementary Table 1. Three-fourths of the patients would continue exercising with the app, and more than half of the patients thought that the app had a positive influence on mobility and pain (Fig. 3: Results Feedback Questionnaire).

**Table 3:** Training adherence of the intervention group (*n* = 30). Self-reported training adherence of the intervention group (*n* = 30) reported after 12 weeks of exercising with ViViRA

How many times have you exercised with the ViViRA app?			
1 times/week	2–3 times/week	4–6 times/week	7 times/week
7% (*n* = 2)	63% (*n* = 19)	20% (*n* = 6)	10% (*n* = 3)

## Discussion

The main results of the present study support our hypothesis that the use of the DHA ViViRA for more than 12 weeks in SpAs patients can improve spinal mobility (by BASMI) and leads to an improvement of self-reported back pain level. Interestingly, the control group showed a worsening in mobility. It is conceivable that participating in the intervention of the study motivated the patients to be more physically active. Furthermore, the DHA covers the functional components of endurance, strength, mobility and neuromotor performance well, which is not always guaranteed with individual physiotherapy. Physiotherapy sessions do not follow a protocol or standardization. Passive muscle movements or massages can also be part of physiotherapy and therefore do not have a major effect on mobility. Mobility improvement is generally greater for females and older participants. This confirms previous findings of a meta-analysis of 26 studies that analyzed the effectiveness of home exercise programs through DHA and reported improvements in lower extremity muscle strength, functional capacity, the number of falls, and the impact on mobility, particularly among older adults, which emphasizes the potential of DHA for home training to improve physical function and decrease disease burden [40].

In both groups, the SF-36 physical score significantly increased (*p* = 0.00), with higher scores for males and younger participants. These results are consistent with a previous clinical study that confirmed that patients with axial spondyloarthropathy (axSpA) improved their quality of life and self-reported health status after one year of participating in a special individualized exercise program [41]. The negative influence of limited mobility and a decline in physical quality of life mainly affects younger patients under the age of 45 with back pain for more than 3 months [42], which is consistent with our study results. These results are also underlined by a systematic review and meta-analysis of a total sample of 15,623 participants with chronic musculoskeletal pain (pain duration > 3 months), which demonstrated the significant associations of pain-related anxiety, fear of pain, and fear-avoidance beliefs with greater pain intensity and disability [43].

An improvement in pain (PAIN-Detect, general pain) was observed in both groups (*p* = 0.00), particularly among females. According to the literature, pain appears to be sex dependent. Female SpAs patients are more likely to have axial (odds ratio 3.3, *p* = 0.01) and peripheral (odds ratio 2.3, *p* = 0.02) pain [44]. It is known that neuropathic pain also occurs in patients with SpAs [45]. Neuropathic pain improves with exercise, such as muscle stretching, muscle strengthening, aerobics, stabilization training, yoga, and Pilates [46]. Whether this improvement is sex specific requires further research.

General pain levels were greater at baseline than at follow-up (*p* = 0.00). It is known, that physical activity (i.e., occupational load and nonoccupational physical activities) reduces the occurrence of back pain [47], and exercise treatment is better than no exercise treatment in the treatment of low back pain [48].

Self-reported back pain levels were greater at baseline for overweight participants in both groups (*p* = 0.03). It is not surprising that obesity appears to have a negative impact on the perception of pain [49], and a significant association of chronic pain with overweight (OR = 1.2, *p* < 0.01) and obesity (OR = 1.8, *p* < 0.01) has been previously shown [50]. In a cross-sectional study with 2509 participants suffering from chronic pain, there was an association between higher BMI and increased pain severity [51].

While the group practicing with ViViRA demonstrated positive outcomes, the physiotherapy group experienced greater improvement in perceived disease activity than did the intervention group (*p* = 0.03). However, the results are difficult to interpret, as not all patients in the control group had started physiotherapy for the first time; in some cases, physiotherapy was continued as before. Studies have shown the effectiveness of physiotherapy with that of home-based exercise programs in patients with SpAs. The reasons given for these results included, among other things, optimal integration through the personal care provided by the physiotherapist, flexible care depending on the physical condition of the day, and good adherence [8].

A disadvantage of exercising at home with ViViRA is certainly the lack of personal support. The patients do not receive feedback when performing the exercises, and there is no control over their adherence to therapy [52]. However, in this study, only 3/30 participants stated that they were missing something such as this. The manageable period of 12 weeks and the motivation through participation in a study certainly played a large part in the participants’ adherence to therapy. The fact that most participants in the intervention group wanted to have the application re-prescribed also speaks for good adherence. A systematic review and meta-analysis study examined at the factors that influence adherence to exercise and demonstrated that the severity of the disease, delay in diagnosis, supervision, and education are relevant influencing factors [53]. More patient education about the positive influence of exercise on symptoms and disease activity increases adherence to exercise [54]. There are already initial research results on how therapy adherence can be increased through the design of applications. For example, as already used with ViViRA, messages with reminders and social support have a positive effect. If this knowledge is used further in the future, adherence can probably be further increased [55].

### Limitations

There were several limitations to this study. For a better analysis of the long-term effects, further studies with longer observation periods are needed [56]. A previous systematic review reported the greatest effect 24 months after physical activity interventions were started [57]. Further studies with a larger number of study participants would better detect and demonstrate differences between the groups [58]. There is also evidence that men and women respond differently to physical activity interventions [59], and in contrast to our results, females usually present with better BASMI scores than males do [60]. The sex distribution in this study does not correspond to the biological sex distribution because there were significantly more females in both groups. One reason may be that women were easier to reach by telephone during recruitment. Another study with a more balanced sex ratio is needed. The time of the functional measurements (BASMI) for the individual patients at baseline and follow up was not kept constant, but the measurements were taken at random times of the day, depending on the clinical appointments of the patients. This could have influenced our outcome measures. Since many patients complain of stiffness in the morning and feel more mobile in the evening, future studies should schedule follow-up at the same time as the baseline visit [61]. The training frequency and intensity were only checked subjectively via questionnaires and personal questions. The objective measurement by the software itself can also be pursued in further studies. Controlling usage by the app provider or tracking it with a fitness bracelet would make usage more objective. In addition, the correct execution of the exercises cannot be guaranteed without a personal coach. A detailed introduction to and explanation of the execution of the exercises would improve patient compliance.

### Conclusion and future directions

In this study, regular exercise therapy via the ViViRA application positively affected mobility and physical function, in patients with SpAs. This study highlights the potential benefits of DHA in the treatment of SpAs, although traditional physiotherapy is also effective, particularly in improving pain. Furthermore, these findings suggest potential for the integration of DHA into routine clinical practice and the management of chronic diseases.

## Electronic supplementary material

Below is the link to the electronic supplementary material.

Supplementary Material 1

## Data Availability

The data sets are available on reasonable request from the corresponding author.

## Abbreviations

ASAS: Assessment of SpondyloArthritis International Society
App: Application
axSpA: Axial spondyloarthropathy
SpAs: Spondyloarthritidess
BASMI: Bath Ankylosing Spondylitis Metrology Index
BMI: Body mass index
CRP: C-reactive protein
DGRh: German Society of Rheumatology
DHA: Digital health application
e.g.: Exempli gratia
HAQ: Health assessment Questionnaire
i.e.: Id est
IQR: Interquartile range
N: Number
OR: Odds ratio
RedCap: Research Electronic Data Capture
SD: Standard deviation
SF: 36-Short Form 36
VRS: Verbal rating scale
WPAI: Work Productivity and Activity Impairment
