# Supplementary material for: Impact of the digital health application ViViRA on spinal mobility, physical function, quality of life and pain perception in spondyloarthritides patients: a randomized controlled trial
Source: Arthritis Res Ther. 2024 Dec 3;26:208. doi: 10.1186/s13075-024-03443-1 (PMC11613898; doi:10.1186/s13075-024-03443-1)
Supplement: Supplementary file 1 — Supplementary Material 1 [file 13075_2024_3443_MOESM1_ESM.docx]

| Questions | Answers | | | | |
| --- | --- | --- | --- | --- | --- |
| How did you find the difficulty of the exercises? | Too easy  3/30  (10%) | Easy  10/30  (33%) | Just right  17/30  (57%) | Difficult  0/30 | Too difficult  0/30 |
| Did you miss someone during the exercises to control your execution and, if necessary, improve it? | Yes  3/30  (10%) | No  22/30  (73%) | Maybe  5/30  (17%) |  |  |
| Do you have the impression that using the app has had a positive effect on your mobility? | Yes  19/30  (63%) | No  1/30  (3%) | Maybe  10/30  (33%) | Don´t know  0/30 |  |
| Do you have the impression that using the app has had a positive influence on your pain symptoms? | Yes  14/30  (47%) | No  2/30  (7%) | Maybe  10/30 (33%) | Don´t know  4/30  (13%) |  |
| Would you continue to use the app outside of the study? | Yes  19/30  (63%) | No  5/30  (17%) | Maybe  6/30  (20%) | Don´t know  0/30 |  |

**Table:** Feedback Questionnaire of the intervention group (n = 30)

Results of the feedback questionnaire of the intervention group (n=30) after 12 weeks of exercising with ViViRA in a randomized controlled trial with 59 axSpA patients (71.2% female, 28.8% male) in Erlangen, Germany, from February 2023 to January 2024.
